# Rice Under Dry Cultivation–Maize Intercropping Improves Soil Environment and Increases Total Yield by Regulating Belowground Root Growth

**DOI:** 10.3390/plants13212957

**Published:** 2024-10-23

**Authors:** Zhihai Wu, Bei Xue, Shiwen Wang, Xu Xing, Min Nuo, Xin Meng, Meikang Wu, Hao Jiang, Huimin Ma, Meiying Yang, Xiaoshuang Wei, Guangxin Zhao, Ping Tian

**Affiliations:** 1 Faculty of Agronomy, Jilin Agricultural University, Changchun 130118, China; wuzhihai@jlau.edu.cn (Z.W.); weixiaoshuang@jlau.edu.cn (X.W.); zhaoguangxin@jlau.edu.cn (G.Z.); 2 Jilin Provincial Laboratory of Crop Germplasm Resources, Changchun 130118, China; 3 College of Life Sciences, Jilin Agricultural University, Changchun 130118, China

**Keywords:** rice under dry cultivation, maize, intercropping, soil fertility, yield

## Abstract

Under the one-season-a-year cropping pattern in Northeast China, continuous cropping is one of the main factors contributing to the degradation of black soil. Previous studies (on maize–soybean, maize–peanut, and maize–wheat intercropping) have shown that intercropping can alleviate this problem. However, it is not known whether intercropping is feasible for maize and rice under dry cultivation, and its effects on yield and soil fertility are unknown. A three-year field-orientation experiment was conducted at Jilin Agricultural University in Changchun city, Jilin Province, China, consisting of three cropping regimes, namely rice under dry cultivation–maize intercropping (IRM), sole rice under dry cultivation (SR), and sole maize (SM). All straw was fully returned to the field after mechanical harvesting. Rice under dry cultivation–maize intercropping with a land-equivalent ratio of 1.05 (the average of three years values) increased the total yield by 8.63% compared to the monoculture system. The aggressivity (A), relative crowding coefficient (K), time–area-equivalent ratio (ATER), and competition ratio (CR) value were positive or ≥1, also indicating that the rice under dry cultivation–maize intercropping had a yield advantage of the overall intercropping system. This is because the intercropped maize root length density (RLD) increased by 33.94–102.84% in the 0–40 cm soil layer, which contributed to an increase in the soil porosity (SP) of 5.58–10.10% in the 0–30 cm soil layer, an increase in the mean weight diameter of soil aggregates (MWD) of 3.00–15.69%, an increase in the geometric mean diameter of soil aggregates (GMD) of 8.16–26.42%, a decrease in the soil bulk density (SBD) of 4.02–7.35%, and an increase in the soil organic matter content (SOM) of 0.60–4.35%. This increased the water permeability and aeration of the soil and facilitated the absorption of nutrients and water by the root system and their transportation above ground, and the plant nitrogen, phosphorus, and potassium accumulation in the intercropping system were significantly higher than that in monoculture treatment, further promoting the total yield of intercropping. This suggests that rice under a dry cultivation–maize intercropping system is feasible in Northeast China, mainly because it promotes belowground root growth, improves the soil environment, and increases the total yield of intercropping.

## 1. Introduction

Since the 20th century, irrational agricultural farming measures have led to a decline in the average thickness of the black soil layer in Northeast China, and black soil degradation, the overdraft of fertility, and thinning and hardening have become significant problems threatening food ecological security [1]. In recent years, 80% of the world’s arable land is rain-fed agriculture, which typically results in lower yield levels [2]. With the continuous expansion of the planting area, the contiguous land area has also been increasing year by year. It has become one of the main factors restricting the further improvement of food production due to lower crop yields, soil crusting, loss of soil nutrients, the frequent occurrence of pests and diseases, and the deterioration in crop quality [3,4]. A complete solution to black soil degradation has not yet been found. However, some techniques can effectively mitigate the extent of black soil degradation, such as changing the cultivation and farming system and carrying out rational diversity planting.

The loss of crop diversity in agricultural practices, including certain planting methods, can significantly disrupt ecosystem balance. Mixed cropping can enhance plant productivity, improve soil nitrate nitrogen absorption, and increase the content of soil organic carbon and plant carbon storage [5]. However, intercropping has gained popularity over mixed cropping due to the short frost-free period and the widespread availability of land suitable for mechanized farming in Northeast China. Intercropping techniques can improve crop yields and quality by optimizing crop combinations and cropping practices [6,7], which have significant potential advantages for boosting system productivity [8]. Intercropping different crops can prevent soil erosion; improve soil fertility; reduce the incidence of diseases, insects, and weeds [9,10,11]; and improve land utilization and ecosystem stability [9]. Among these, the interaction of the root distribution of intercropping species in the subsurface plays a key role in resource utilization and yields an advantage in intercropping systems [12]. It has been reported that intercropping root overlap fully utilizes underground resources, resulting in a higher root mass of intercropped species than that found in monocropping [13]. The multi-interface interactions of crops exist in crop diversity systems, including plant–plant, plant–soil, and aboveground–belowground interactions, which can be strategically leveraged to enhance nutrient-use efficiency and boost crop productivity [14]. Intercropping absorbs more soil water and nutrients through better root proliferation and interspecific root interactions. Intercropping cereals with legumes is a widely practiced agricultural strategy across many countries [15], which improves soil fertility, reduces weeds and insect pests, and increases yields [16]. In a maize–soybean intercropping system, maize serves as the advantage crop, which reduces its competitive ability while significantly enhancing that of the soybean, significantly improving the overall advantages of intercropping [17]. In addition, Guo et al. (2021) and Zheng et al. (2022) showed that a wheat–maize-soybean relay strip intercropping system can increase the soil organic matter content and organic matter fractions, improve the soil physical structure, and enhance soil microbial community diversity [18,19].

Maize–peanut intercropping can increase the availability of soil-effective phosphorus [20]. Intercropping increased the soil water content in maize rows during the drumming stage, leading to improved water-use efficiency for crop growth, which enhanced the grain weight of maize while reducing both the grain weight and the number of pods per soybean plant [21]. The most common mode of intercropping cereals with cereals is maize–wheat, which has been a prevalent agricultural practice in Northwest China, and the method has been shown to be effective, with land-equivalent ratios commonly exceeding one, indicating that this intercropping system can yield more than the sum of individual crop yields [22,23]. The significantly decreased radiation-use efficiency of maize demonstrated that wheat was the advantageous crop in this intercropping mode [8].

Maize and rice are the main food products in Northeast China, while rice production is encountering significant challenges due to water scarcity. Water-saving cultivation of rice, including wetting and drying irrigation, controlled irrigation, and rice under dry cultivation have been considered appropriate cultivation technique measures to cope with the difficult problem in China [24,25]. Meanwhile, rice under dry cultivation can be directly sown under dryland conditions and grown in low-lying and flood-prone land, which improves land-use efficiency [26,27]. The advantages of intercropping have been confirmed in previous studies. The rice under dry cultivation is shallow rooted crops and maize has a deep root system, the differences in spatial structure between aboveground and belowground and their interactions has been followed with interest. However, the feasibility of rice under dry cultivation–maize intercropping and its impact mechanisms on crop yield and soil fertility are still unclear. Therefore, the objectives of this study were (1) to clarify the effect of rice under dry cultivation–maize intercropping on the crop yield and distribution of roots in the subsoil; (2) to investigate the effect of rice under dry cultivation–maize intercropping on the physicochemical properties of the soil and the nutrient of plant; and (3) to analyze the relationship between the distribution of roots in the subsoil of rice under dry cultivation–maize intercropping and the physicochemical properties of the soil. This study provides new cultivation measures to protect the healthy development of green and sustainable agriculture in the black soil and drylands of Northeast China.

## 2. Result

### 2.1. Yield and Land-Use Efficiency

The average total yield of intercropping was 8.64% higher than that of monocropping over the 3 years (Table 1). The PLER_R_ values were 0.43, 0.46, and 0.47, and the PLER_M_ values were 0.63, 0.61, and 0.56 for the 3-year period. The average land-equivalent ratio was 1.05, which is greater than 1. The average yield of the intercropped rice under dry cultivation was 6417.78 kg ha^−1^ over the 3 years, which is 9.23% lower than that of the SR (7070.42 kg ha^−1^). The average yield of the intercropped maize was 12,927.45 kg ha^−1^, which is 20.40% higher than that of the SM (10,737.39 kg ha^−1^). The average number of ears per square meter (EN) of intercropped rice under dry cultivation over the 3 years was 2.81% lower than that of the SR (Table 2). The number of grains per spike (KN) and the thousand-grain weights (TKWs) of the intercropped maize increased by 3.95% and 4.07%, respectively, compared to the SM. The yields of rice under dry cultivation were not affected by year, in contrast to the maize yields, which were significantly affected by year. The cropping system drastically affected the yields of rice under dry cultivation and maize, and the interaction of the cropping system in the years had no effect on the yield of the rice under dry cultivation or the maize.

### 2.2. Time–Area-Equivalent Ratios, Aggressivity, Relative Crowding Factors, and Competition Ratios

Over the three years, the A_M_, A_R_, and A_MR_ values were positive, which indicates that maize was the dominant species in the rice under the dry cultivation–maize intercropping system. The K_R_ value was <1, indicating that the rice under dry cultivation has a yield drawback in the intercropping system, while the K_MR_ value was ≥1 over the three years, indicating that the rice under dry cultivation–maize intercropping had a yield advantage of the overall intercropping system. The ATER values were >1 over the three years, indicating the existence of a yield advantage for the rice under dry cultivation–maize intercropping system. The CR_MR_ was >1 over the three years, indicating that maize had higher competitive intensity relative to rice under dry cultivation when intercropped (Table 3).

### 2.3. Aboveground Nutrient Accumulation

The rice under dry cultivation–maize intercropping significantly decreased the accumulation of N, P, and K in the intercropped rice under dry cultivation and increased the accumulation of N, P, and K in the intercropped maize within 2 years (Table 4). The aboveground nitrogen accumulation of the intercropped rice under dry cultivation was an average of 14.40% lower than that of the monoculture. The aboveground phosphorus accumulation was an average of 28.04% lower than that of monoculture, and the aboveground potassium accumulation was an average of 24.59% lower than that of monoculture. On the contrary, the aboveground nitrogen accumulation of the intercropped maize increased by an average of 19.82%. The aboveground phosphorus accumulation by an average of 25.73%, and the aboveground potassium accumulation by an average of 16.91% compared to monocropping. This suggests that rice under dry cultivation–maize intercropping can significantly promote the accumulation of nutrients in maize aboveground.

### 2.4. Root Growth and Distribution

**Figure 1:**
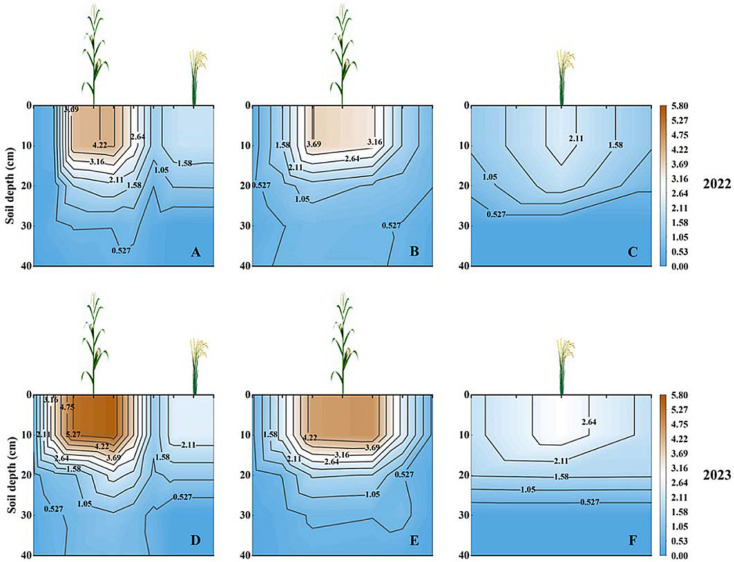
Two-dimensional root length density (cm cm^−3^) distribution at a 0–40 cm soil depth in the filling growth stages in different planting systems. (**A**,**D**) Rice under dry cultivation–maize intercropping; (**B**,**E**) sole maize; (**C**,**F**) sole rice under dry cultivation.

Different cropping systems significantly affected root growth and distribution in both crops (Figure 1). The total RLD in the 0–40 cm soil layer of the intercropped maize was increased by 26.43% compared to the SM (Figure 1A,B,D,E). Horizontally, the maize root system in the intercropping system extended toward the rice in the dry cultivation rows (Figure 1A,D), indicating that the maize root system was widely distributed in the rice under the dry cultivation rows and absorbed water and nutrients during the filling period. The roots at different sampling locations and in different years were mainly concentrated within a depth of 0–30 cm, accounting for 91.14% of the total RLD. The total RLD was reduced by 12.6% in the intercropped rice under dry cultivation in the 0–40 cm soil layer compared to the SR (Figure 1A,C,D,F). In the horizontal direction, the roots of intercropped rice under dry cultivation were present only in the rows of rice under dry cultivation. This suggests that intercropping promotes maize root growth to the detriment of rice under dry cultivation.

### 2.5. Soil Physical and Chemical Characteristics

**Figure 2:**
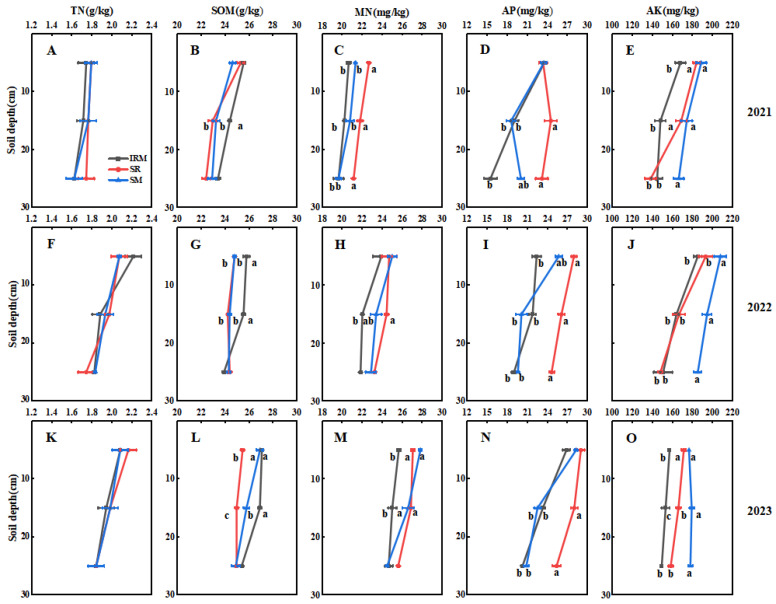
Soil total nitrogen (TN), soil organic matter (SOM), soil mineral nitrogen (MN), soil-available phosphorus (AP), and soil-available potassium (AK) in rice under dry cultivation and maize intercropping and monocropping systems at soil depths of 0–30 cm in 2021–2023. Different lowercase letters indicate differences (*p* < 0.05) amongst cropping patterns in the same soil layer. (**A**,**F**,**K**) soil total nitrogen, (**B**,**G**,**L**) soil organic matter, (**C**,**H**,**M**) soil mineral nitrogen, (**D**,**I**,**N**) soil-available phosphorus, (**E**,**J**,**O**). Sole rice under dry cultivation (SR), sole maize (SM), rice under dry cultivation–maize intercropping (IRM).

The effect of rice under dry cultivation–maize intercropping on the soil nutrients was significant (Figure 2). The soil nutrients increased as the experimental years increased, and consistent changes were observed across the three treatments each year. The TN of intercropping was not significantly different from monocropping within the 0–30 cm soil layer in 2021–2023 (Figure 2A,F,K). The SOM of intercropping increased by 7.74% and 4.35% compared to the SR and SM, respectively, in the 10–20 cm soil layer (Figure 2L). The MN of intercropping decreased by 6.94% and 5.99% compared to the SR and SM, respectively, in the 10–20 cm soil layer (Figure 2M). The AP of intercropping decreased by 23.00% compared to the SR 10–20 cm soil layer (Figure 2N). The AK of intercropping decreased by 7.91% and 14.66% compared to the SR and SM, respectively, in the 10–20 cm soil layer (Figure 2O), and this trend was consistent throughout the seasons of 2021–2023. This suggests that rice under dry cultivation–maize intercropping can significantly increase the SOM in the 10–20 cm soil layer and promote the uptake and utilization of soil-effective nutrients by aboveground plants (Table 4).

**Figure 3:**
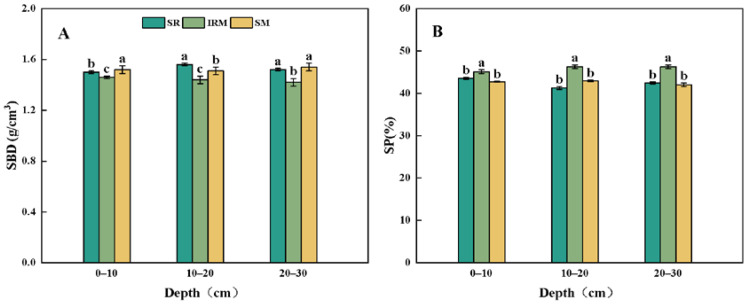
Soil bulk density (SBD) and soil porosity (SP) in rice under dry cultivation and maize intercropping and monocropping systems at a soil depth of 0–30 cm. Different lowercase letters above bars indicate significant differences (*p* < 0.05) amongst cropping patterns in the same soil layer. (**A**) Soil bulk density, (**B**) soil porosity. Sole rice under dry cultivation (SR), sole maize (SM), rice under dry cultivation–maize intercropping (IRM).

Rice under dry cultivation–maize intercropping had a significantly lower SBD and a higher SP (Figure 3). In the 0–10 cm, 10–20 cm, and 20–30 cm soil layers, the SBD of the IRM decreased by 4.02%, 4.58%, and 7.35% (Figure 3A), and the SP increased by 5.58%, 7.76%, and 10.10% compared to the SM, respectively (Figure 3B). This indicates that intercropping was effective in reducing the SBD, improving the SP, and increasing the soil water permeability and aeration in the 0–30 cm soil layer compared to monocropping.

**Figure 4:**
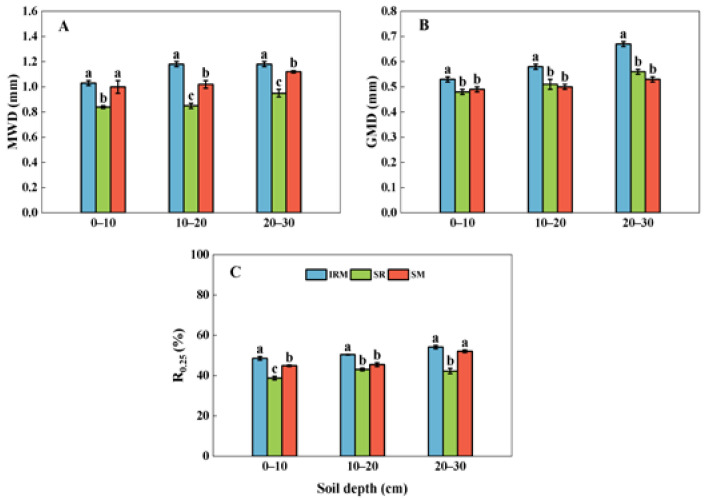
Mean weight diameter (MWD), geometric mean diameter (GMD), and percentage of >0.25 mm aggregate (R_0.25_) in rice under dry cultivation and maize intercropping and monocropping systems at soil depths of 0–30 cm. Different lowercase letters above bars indicate differences (*p* < 0.05) amongst cropping patterns in the same soil layer. (**A**) Mean weight diameter, (**B**) geometric mean diameter, (**C**) percentage of >0.25 mm aggregate. Sole rice under dry cultivation (SR), sole maize (SM), rice under dry cultivation–maize intercropping (IRM).

As can be seen in Figure 4, the MWD, GMD, and R_0.25_ were significantly higher under intercropping than monocropping. Compared to the SM, the MWD of the IRM increased by 15.69% and 5.36% in the 10–20 cm and 20–30 cm soil layers (Figure 4A), the GMD increased by 8.16%, 16.00%, and 26.42% in the 0–10 cm, 10–20 cm, and 20–30 cm soil layers (Figure 4B), and the R_0.25_ increased by 8.20% and 10.89% in the 0–10 cm and 10–20 cm soil layers (Figure 4C), respectively. The above results indicate that intercropping improved the stability of the soil aggregates in the 0–30 cm soil layer.

### 2.6. Principal Component Analysis

**Figure 5:**
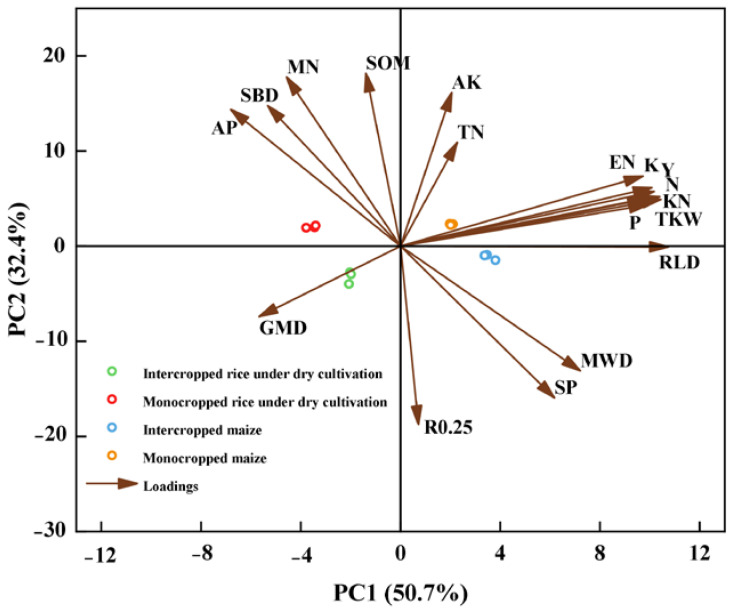
Principal component analysis (PCA) among yield, yield components, root distribution, and physical and chemical characteristics of soil under rice under dry cultivation and maize in intercropping and monoculture systems in 2023. Y, yield; EN, number of ears per square meter; KN, number of grains per spike; TKW, thousand-grain weight; N, total nitrogen content of plants; P, total phosphorus content of plants; K, total potassium content of plants; RLD, root length density; SBD, soil bulk density; SP, soil porosity; MWD, mean weight diameter of aggregates; GMD, geometric mean diameter of aggregates; R_0.25_, soil aggregate ratio greater > 0.25 mm; TN, soil total nitrogen; SOM, soil organic matter; MN, soil mineral nitrogen; AP, soil-available phosphorus; AK, soil-available potassium.

Principal component analysis (PCA) was carried out for 2023 to reveal the relationships among the yield and yield components, root distribution, and soil physicochemical properties under intercropping and monocropping (Figure 5). Two principal components (PCs 1 and 2) were chosen, which explained 50.7% and 32.4% of the variation, respectively. The correlation between the smaller acute (<90°) load vectors was stronger. This shows that the yield (Y) was significantly positively correlated with the EN, KN, TKW, N, P, K, RLD, MWD, and SP and significantly negatively correlated with the SBD. RLD was significantly and positively correlated with MWD, SP, and R_0.25_.

## 3. Discussion

### 3.1. Yield Performance and Land-Use Efficiency

The net predominant productiveness of terrestrial ecosystems usually increases with biodiversity [28]. Due to the temporal and spatial differences in ecological areas of interest, the intercropping of multiple species enhances productivity through the efficient use of water, temperature, and light resources [29]. In this research, intercropping reduced the yield of rice under dry cultivation but elevated the yield of maize, and the increase in maize yield was greater than the decline in rice yield under dry cultivation. As a result, the total yield of the rice under dry cultivation–maize intercropping was greater than that of the weighted monocropping (Table 1). The LER was greater than one in all three years (Table 1), indicating that the rice under dry cultivation–maize intercropping improved the land-use effectively and that intercropping extended food manufacturing without enhancing the area of cultivated land. Previous studies have also tested the yield and land-use advantages of intercropping, with combinations such as wheat–maize, peanut–maize, soybean–maize, and the increase in yield from intercropping systems can be primarily attributed to the spatial distribution of aboveground biomass, which enhances the productivity of advantageous crops and improves the LER [8,17,22,30]. The increase in biomass is primarily driven by two key factors. Around two-thirds of the contribution comes from the border-row effect, while one-third is attributed to the inner-row effect [22]. Adjusting the row ratios of disadvantage crops to optimize both aboveground and belowground spatial configurations further enhances the overall yield of the system [8,17]. The yield of intercropped rice under dry cultivation was drastically lower than that of the SR (Table 1), which was due to the reduced EN and KN of the intercropped rice under dry cultivation compared to the SR (Table 2) and the competitive disadvantage of the intercropped rice under dry cultivation during the growth period. Therefore, the root growth of the intercropped rice under dry cultivation was hindered, and the absorption of the soil water and nutrients was lower, which was unfavorable for the growth and yield formation of the rice under dry cultivation. However, the yield of the intercropped maize was greater than that of the SM (Table 1) due to the notably greater KN and TKW of the intercropped maize than the SM (Table 2). A previous study proved that the dry matter of intercropped maize substantially multiplied, promoting a maize kernel boom and increasing the maize yield [17]. This suggests that there is a total yield advantage of intercropping rice under dry cultivation and maize.

The results were further validated by analyzing the competition between the two crops under different treatments. In the system of intercropping, interspecific competition and facilitation are the two usual coexistence interactions [31]. Facilitation occurs when one crop accelerates the growth of any other crop and has been determined in most cereal–legume cropping systems. In contrast, interspecific opposition takes place when one crop limits the growth or yield of another [32,33]. In this study, the rice under dry cultivation–maize intercropping showed strong interspecific competition during the symbiotic period. The positive A_MR_ values over the 3-year period indicate that maize was the dominant species to the rice under dry cultivation–maize intercropping system, and the rice under dry cultivation was the weaker species. The K_M_ values were all greater than one, while the K_R_ values were less than one, indicating that maize is an advantageous crop, whereas rice under dry cultivation is a disadvantaged crop within the intercropping system. Furthermore, the K_MR_ values were all equal to or greater than one, suggesting that this system effectively enhances production. The CR_MR_ was >1, indicating a greater competitive intensity of the maize and a lesser competitive intensity of the rice under dry cultivation under intercropping (Table 3), which may be because the ecological niche is better for taller maize than for shorter rice under dry cultivation. These results are in line with the findings of Raza et al. [34] on intercropping, showing that the dominance of taller crops in intercropping resulted in capturing more resources in the long term, which in turn yielded higher grain yields.

### 3.2. Root Characteristics

The plant root system is a key place for subsurface interspecies interactions because the roots chiefly obtain water and nutrients, making them accessible to different tissues of the plant [22]. In addition to aboveground interactions, belowground interactions consisting of the root distribution of intercropped species also play a key function in identifying resource utilization and yield advantages in intercropping structures [12]. Interspecific promotion changes the ecology of one crop in favor of another [35]. Such root interactions between intercrops appear more frequently when two plants are intercropped [36]. The RLD of the intercropped maize was significantly higher than that of the SM, but the RLD of the intercropped rice under dry cultivation was significantly lower than that of the SR. And the root system of the intercropped maize extended horizontally to the rows of the dry-cropped rice and vertically to a depth of 40 cm (Figure 1). This suggests that the rice under dry cultivation–maize intercropping promoted the root growth of maize, which facilitates the aboveground plant’s access to nutrients and water, and ultimately increased yields. A previous study also reported that intercropped species are superior to monocropped species in accessing water and nutrients [37]. This interaction, which can additionally be defined as uneven interspecific facilitation, resulted in maize outperforming rice under dry cultivation in terms of root growth, nutrient uptake, and yield.

### 3.3. Soil Physical and Chemical Properties

Soil nutrients are one of the significant limiting factors of yield. Intercropping can improve fertility by helping to improve nutrient availability and soil quality [38,39]. In this study, we compared the changes in the soil physicochemical properties between intercropping and monocropping for three years and found that the rice under dry cultivation–maize intercropping significantly increased the SOM in the 10–20 cm soil layer (Figure 2B,G,L) and promoted the uptake and utilization of soil effective nutrients by aboveground plants (Table 4). Although the TN of intercropping did not differ from that of monocropping, the TN (1.95 g kg^−1^) increased after 3 years compared to the initial value (1.43 g kg^−1^) before the beginning of the experiment (Figure 2A,F,K), and the MN and AP showed the same trend (Figure 2C,D,H,I,M,N), suggesting that this intercropping system maintains soil fertility over a longer period of time. Returning straw to fields accelerates the growth and reproduction of soil microorganisms due to the release of nutrients from straw decomposition, enriching maize root secretions with nutrients such as amino acids [40,41,42]. This increases the active carbon and nitrogen of the inter-root soil, among other benefits. Therefore, the above findings are worthy of further study.

SBD and SP are key indicators that characterize the physical properties of soil, and the impact of different cropping practices on soil physical properties varies due to the regional soil type, years of tillage management, and mechanical operations [43]. In this study, it was found that the rice under dry cultivation–maize intercropping significantly reduced the SBD and increased the SP (Figure 3). Studies on root growth have shown that roots from different crops cross soil pores in different ways [44,45], with the roots of small grains, such as barley and wheat, able to cross soil macropores. And the roots of maize or soybeans are able to grow in macropores and reenter the bulk of the soil in the tillage layer [46]. Due to the well-developed root system of maize, the extension area is wider, resulting in a lower SBD and higher SP, which enables the absorption of more water and nutrients [47]. Soil aggregates are the basic units of soil structure and serve as habitats for soil microorganisms and nutrient storage sites [48]. The soil aggregate content and particle size distribution not only affect crop increase and development, but they also have an essential impact on a series of physical, chemical, and organic properties of soil. The structural stability of soil aggregates can be expressed in terms of the MWD and GMD of soil aggregates. The higher the MWD and GMD of the soil aggregates, the stronger the aggregate stability [49]. It was confirmed via a long-term localization experiment that intercropping unique vegetation increased the soil macroaggregate content and improved the stability of aggregates [50]. On one hand, this is due to the increase in the type and quantity of root secretions in diversified planting patterns and the increase in the vitality of inter-root fungi and mycelium, which promotes the growth of mycelium and the secretion of polysaccharides and enhances the gelling effect of micro-aggregates, thus promoting the transformation of micro-aggregates into macro-aggregates and enhancing the stability of soil agglomerates [51,52]. On the other hand, the alternation of different crops is conducive to the maintenance of soil biodiversity, and soil carbon and nitrogen transformations are enhanced, producing large amounts of proteins, polysaccharides, lignin, and other substances, which indirectly promote the formation of macroaggregates [53,54]. In addition, diversified cropping (intercropping and crop rotation) patterns enhance soil aggregation by indirectly affecting the soil microbial community composition, such as by increasing the relative abundance of soil Sordariales and Ascomycetes fungi or decreasing the relative abundance of Nitrospirae [55], which indirectly contributes to the formation of soil aggregates [56]. In this study, we showed that, compared to the SM, the rice under dry cultivation–maize intercropping increased the content of large soil water-stable aggregates (>0.25 mm) and decreased the content of microaggregates (<0.053 mm). Additionally, the rice under dry cultivation–maize intercropping increased the MWD and GMD of the soil aggregates, and the structural stability of the soil aggregates was higher than that under the SM (Figure 4).

**Figure 6:**
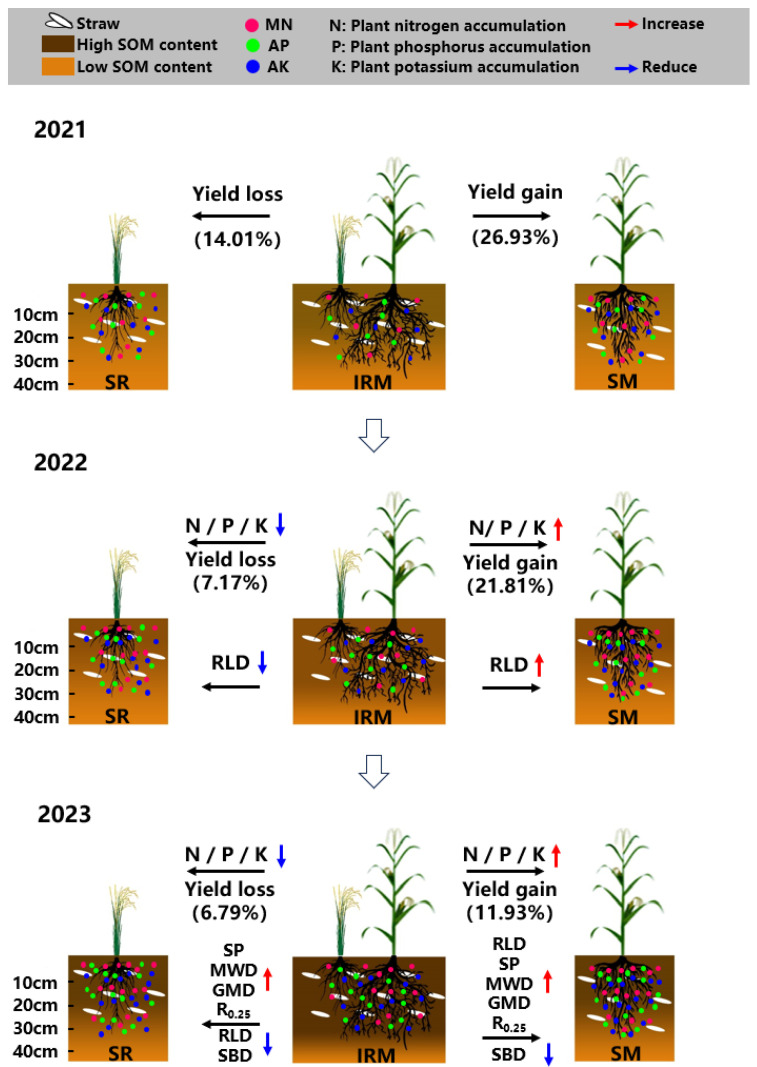
The effects of different planting patterns on yield and soil fertility in 2021–2023. SR, sole rice under dry cultivation, SM, sole maize, IRM, rice under dry cultivation–maize intercropping, SP, soil porosity, MWD, mean weight diameter of aggregates, GMD, geometric mean diameter of aggregates, R_0.25_, soil aggregate ratio greater > 0.25 mm, SBD, soil bulk density, RLD, root length density.

The rice under dry cultivation–maize intercropping system was able to promote maize root growth, improve the soil physicochemical properties, and increase the yield (Figure 6). This is because rice under dry cultivation is small-rooted and maize is large-rooted, and different root types are able to pass through soils with different granular structures, improving soil aeration and water permeability. This helps the root system absorb nutrients and water from the soil and improves the accumulation of nutrients (N, P, and K) in the aboveground plant (Table 4), further increasing crop yields. Intercropped rice under dry cultivation is a disadvantaged crop with stunted root growth and reduced nutrient (N, P, and K) accumulation in the aboveground plant (Table 4), resulting in reduced yields. However, the average increase in the yield of maize (20.22%) in this intercropping system over 3 years was greater than the decrease in the yield of rice under dry cultivation (9.32%). In the future, we should focus on studying how to improve the yield of rice under dry cultivation and the mechanisms of soil nutrient changes.

## 4. Materials and Methods

### 4.1. Site Description

**Figure 7:**
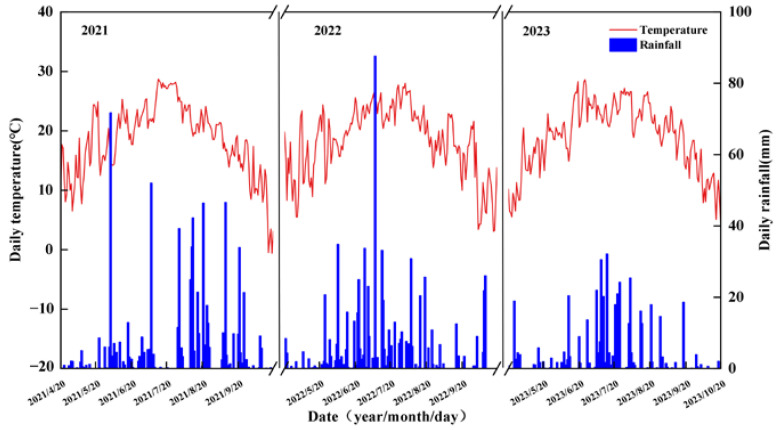
Daily air temperature and daily rainfall during the 2021, 2022, and 2023 growth seasons at the experimental site.

The experiment in the field took place between 2021 and 2023 at the research center of Jilin Agricultural University in Changchun, located in Jilin Province, China (43°81′ N, 125°40′ E). This area has a humid continental climate and experienced average temperatures of 18.45 °C, 18.34 °C, and 18.96 °C over the three-year period, along with annual precipitation levels of 714.8 mm, 697.7 mm, and 465.8 mm, respectively. The chemical parameters of the soil layer at a 0–30 cm depth before planting in 2021 were as follows: the total nitrogen content was 1.43 g kg^−1^; the mineral nitrogen content was 98.90 mg kg^−1^; the available phosphorus content was 28.71 mg kg^−1^; the available potassium content was 161.32 mg kg^−1^; the organic matter content was 20.89 g kg^−1^; and the soil pH was 6.90. Figure 7 displays the rainfall and temperature data at the test site during the growing seasons of 2021, 2022, and 2023.

### 4.2. Experimental Design

Over a three-year period, a trial was carried out using a randomized complete block design, which included three treatments and three replications for each treatment. The three treatments were sole rice under dry cultivation (SR), sole maize (SM), and rice under dry cultivation–maize intercropping (IRM). Each test plot was 10 m by 4.8 m in size, with a 1 m separation zone between adjacent plots. Each intercropping strip consisted of 16 rows of rice under dry cultivation (a 4.8 m wide strip) and 8 rows of maize (a 4.8 m wide strip). Thus, each intercropping plot consisted of 50% rice under dry cultivation and 50% maize. The varieties of rice under dry cultivation and maize used in this study were ‘*Oryza sativa* L. cv. Suiijing18’ and ‘*Zea mays* L. cv. Jinongda935’, respectively, which are commonly used by local farmers. The rice under dry cultivation and maize were sowed on 1 May 2021, 29 April 2022, and 1 May 2023 and were harvested on 2 October 2021, 1 October 2022, and 5 October 2023. After mechanical harvesting, the straw was fully returned to the field with deep tilling (30 cm). The rice under dry cultivation was sown using a hole-sowing method at 150 kg ha^−1^, with a row spacing of 30 cm for both intercropping and monocropping. The density of the maize was 55,500 plant ha^−1^, with 60 cm row spacing and 30 cm plant spacing. The same row spacing was used for intercropping and monocropping. In the intercropping plots, the distance between adjacent rows of rice under dry cultivation and maize was 45 cm.

For rice under dry cultivation (monoculture and intercropping), nitrogen, phosphate, and potassium fertilizer were used, i.e., urea (46% N), calcium phosphate (12% P_2_O_5_), and potassium sulfate (60% K_2_O), with an application rate of 140 kg ha^−1^ for N and 75 kg ha^−1^ for both P_2_O_5_ and K_2_O. The ratio for applying the nitrogen fertilizer was 4:3:3 for the basal fertilizer, tiller fertilizer, and spike fertilizer, respectively. Phosphate and potassium fertilizer, on the other hand, were applied solely as the basal fertilizer. For the maize (monoculture and intercropping), the fertilizer was a blend (N-P_2_O_5_-K_2_O: 25-10-13), with N, P_2_O_5,_ and K_2_O applied at 225 kg ha^−1^, 90 kg ha^−1^, and 117 kg ha^−1^, respectively, with a one-time application of the full basal fertilizer. The other management was based on local traditional high-yield cultivation practices.

### 4.3. Measurement Index and Methods

#### 4.3.1. Yield Measurement

At the maturity stage, the intercropped rice under dry cultivation and maize were sampled by row, and the rice under dry cultivation was taken from 8 rows near the side. Effective spikes from a total of 25 holes were investigated for each row, and 10 holes of the representative rice under the dry cultivation plants were selected from 10 holes, in accordance with the average number of effective spikes. Then, the spikes were naturally air-dried for the determination of the number of grains per spike, the weight of 1000 grains, and the rate of fructification. Select 1 m^2^ per row and choose a total of 8 rows as the yield measurement area to be harvested for yield determination. The rice under dry cultivation weight and moisture content were determined after sun-drying, and the averages were calculated based on standardized grain yields determined at a 13.5% moisture content. The maize was taken from 4 rows for yield determination, i.e., 10 m per row. The collected samples were put into gauze mesh bags, which were placed in an air-drying shed until a constant weight was reached to determine the length of the ear, the number of grains on the ear, the depth of the grains, and the weight of 100 grains. The seeds were weighed, and the moisture content was determined using a moisture meter. This was converted to yield at a 14% moisture content, and the averages were calculated. The sole rice under dry cultivation and the sole maize were measured in the middle of the monocropping plots, and their yields were measured in the same way as those in the intercropping plots.

The calculated total yield was determined by averaging the weight of the rice under dry cultivation and the maize grain yield for both intercropping and monocropping [33,57], as shown below:

(1) $$Intercropping\;total\;yield=Y_{R,I}{\;\times \;Z}_{R}+Y_{M,I}{\;\times \;Z}_{M}$$

(2) $$Monocropping\;total\;yield=Y_{R,S}{\;\times \;Z}_{R}+Y_{M,S}{\;\times \;Z}_{M}$$

The yields of the intercropped rice under dry cultivation and maize are denoted as Y_R,I_ and Y_M,I_, respectively, while the yields of the monocropped rice under dry cultivation and maize are represented by Y_R,S_ and Y_M,S_, respectively. Additionally, Z_R_ and Z_M_ indicate the planting proportions of the intercropped plots.

#### 4.3.2. Land = Equivalent Ratio

The calculation of the land-equivalent ratio (LER) was employed to evaluate the benefits of intercropping and was calculated as follows [58]:

(3) $$LER={PLER}_{R}+{PLER}_{M}=\frac{Z_{R}{\;\times \;Y}_{R,I}}{Y_{R,S}}+\frac{Z_{M}{\;\times \;Y}_{M,I}}{Y_{M,S}}$$

PLER_R_ and PLER_M_ are partial LERs for the rice under dry cultivation and maize. Y_R,I_ and Y_M,I_ represent the yields of rice under dry cultivation and maize when intercropped, while Y_R,S_ and Y_M,S_ indicate the yields of rice under dry cultivation and maize when grown separately. Z_R_ and Z_M_ denote the planting proportions in the intercropped field. An LER above 1.0 suggests that intercropping enhances the growth and yield of the crops, while values below 1.0 suggest that intercropping has a detrimental impact on the growth and yield of the crops.

#### 4.3.3. Competition Indices

Various competitive indicators were utilized to calculate the competitive and beneficial impacts of rice under dry cultivation–maize intercropping. The index of aggressivity (A) was utilized to denote the level of competitiveness between the two crops in the practice of intercropping, as stated by Dhima et al. [58]. It was determined using the following formula:

(4) $$A_{M}=\frac{Y_{M,I}}{Y_{M,S}\;\times \;Z_{M}}-\frac{Y_{R,I}}{Y_{R,S}{\;\times \;Z}_{R}}$$

where Y_R,I_ and Y_M,I_ are the yields of the intercropped rice under dry cultivation and maize, respectively; Y_R,S_ and Y_M,S_ are the yields of the monocropped rice under dry cultivation and maize, respectively; and Z_R_ and Z_M_ are the planting proportions of the intercropped plots. If A_M_ = 0, this suggests that the competitiveness of the maize and rice under dry cultivation were equal. If A_M_ > 0, this indicates that the maize was more competitive than the rice under dry cultivation. If A_M_ < 0, this suggests that the maize was less competitive than the rice under dry cultivation.

The crowding coefficient relative (K) is a measure of the predominant species’ relative dominance over another in intercropping [59]. It was determined in the following manner:

(5) $$K=K_{R}{\;\times \;K}_{M}$$

(6) $$K_{R}=\frac{Y_{R,I}{\;\times \;Z}_{R}}{Y_{R,S}\;\times \;Z_{R}}/\frac{Y_{R,S}}{Y_{R,I}}$$

(7) $$K_{M}=\frac{Y_{M,I}{\;\times \;Z}_{M}}{Y_{M,S}{\;\times \;Z}_{M}}/\frac{Y_{M,S}}{Y_{M,I}}$$

If K > 1, this suggests a yield benefit. If K = 1, this suggests no yield benefit, and if K < 1, this suggests a yield drawback.

The competition ratio (CR) is another index to evaluate the degree of competition between one crop and another in intercropping [60]. The specific calculations are as follows:

(8) $${CR}_{MR}=\frac{Y_{M,I}/(Y_{M,S}{\;\times \;Z}_{M})}{Y_{R,I}/(Y_{R,S}{\;\times \;Z}_{R})}$$

where CR_MR_ is the competitive ratio of the maize relative to the rice under dry cultivation. When CR_MR_ > 1, it indicates that the maize was more competitive than the rice under dry cultivation in the intercropping system.

The area–time equivalence ratio (ATER) provides a more accurate comparison of the yield advantage between intercropping and monocropping by considering the time devoted to each component crop within an intercropping system [61]. It is calculated as follows:

(9) $$ATER=\frac{1}{T}\sum_{1}^{n}\frac{dY_{M,I}}{Y_{M,S}}$$

where ‘n’ represents the number of crops involved, ‘d’ signifies the growth period of each crop in days, and ‘T’ denotes the total time in days for which the field remained occupied, corresponding to the growth period of the longest-duration crop. Productivity can also be expressed in terms of the resource-use efficiency of the most limiting resource, such as water, nutrients, energy, or farmland, when t = d in comparison. The numerical values of ATER approach those of LER for a mixture of crops with roughly identical growth periods. ATER > 1 indicates a yield benefit. ATER = 1 indicates no intercropping effect, and ATER < 1 indicates a yield disadvantage.

#### 4.3.4. Plant Nutrient Determination

At maturity, the rice under dry cultivation and maize were killed in an oven at 105 °C, dried at 80 °C, and pulverized in a small pulverizer after being broken down into stems, leaves, and ears with scissors. After the pulverized samples were decocted with H_2_SO_4_-H_2_O_2_, the total nitrogen content was determined using Kjeldahl nitrogen determination. The total phosphorus content was determined using the ammonia vanadium molybdate colorimetric method, and the total potassium content was determined using the flame photometric method [62].

#### 4.3.5. Root Length Density

Root sampling of the rice under dry cultivation and the maize was conducted using the profile method [63] during 2022–2023. Before sampling, the aboveground plants in the selected area were removed. Four 15 cm × 7.5 cm × 10 cm rectangular soil blocks were dug for the rice under dry cultivation, and six 30 cm × 10 cm × 10 cm rectangular soil blocks were dug for the maize to a depth of 40 cm below the ground level, with every 10 cm serving as a layer, for a total of four layers. Then, the soil clods were placed in a 100-mesh nylon mesh bag and rinsed with water. After the soil was washed away, the root system remaining in the mesh bag, along with sand and other impurities, was poured into a pot, and the roots were picked out with forceps. An Epson V700 scanner (Seiko Epson Corp, Nagano Prefecture, Japan) was used to scan images of the acquired roots, and WinRHIZO 2013 (Regent Instruments, Québec City, QC, Canada) was used to calculate the root length (cm).

(10) $$RLD=\frac{RL}{V}$$

where RLD is the root length density (cm cm^−3^), RL is the root length (cm) in a given amount of soil mass, and V is the soil volume (cm^3^).

#### 4.3.6. Soil Samples

Soil sample collection was carried out in the maturity duration of 2021–2023. The soil samples were collected from 0 to 30 cm using a five-point sampling approach. A soil extraction auger with an internal diameter of 5 cm was used to sample in layers of 10 cm, with three replications for every treatment. After thorough mixing, the more obvious straw and grit were removed from the samples, which were then put into Ziploc bags and taken back to the laboratory. The samples were naturally air-dried, ground, and passed through 0.149 mm and 1 mm sieves prior to subsequent determination. The soil mineral nitrogen (MN) was determined by means of an automated intermittent chemical analyzer (Smart Chem 200, Rome, Italy). The soil total nitrogen (TN) was determined using Kjeldahl nitrogen determination. The soil organic matter (SOM) was determined by the volumetric method of potassium dichromate with external heating. The soil-available phosphorus (AP) was determined with the sodium bicarbonate-leaching molybdenum antimony colorimetric method, and the soil-available potassium (AK) was determined using the ammonium acetate-leaching flame photometric technique [57].

At harvest in 2023, in situ samples were collected at each sampling site from 0 to 30 cm in 10 cm layers, with three replicates per treatment using a 100 cm3 ring knife at the sampling locations to determine the soil bulk density (SBD) and the soil porosity (SP) of each layer using the following formulas:

(11) $$SBD=\frac{M_{D}}{V}$$

(12) $$SP=\left(1-\frac{SBD}{SG}\right)\times 100\%$$

where MD denotes the weight of the dry soil (g), and V denotes the cutting ring volume (100 cm^3^). SG stands for specific gravity, where the SG is approximated as 2.65 g cm^−3^.

The soil samples were taken from the 0–30 cm depth in layers of 10 cm for the rice under dry cultivation and at maize maturity in 2023, with three replications for each treatment. The intercropping treatment was sampled by collecting two random points, namely from beneath the rice under the dry cultivation belt and the maize belt, and mixing them into one soil sample.

The soil aggregates were determined using the moist sieving method by putting the samples on successive sieve units with pore sizes of 2, 1, 0.5, and 0.25 mm from largest to smallest and sieving them with oscillation for 5 min (30 times min^−1^). Then, the aggregates were subsequently washed from each sieve layer into an aluminum box, dried, and weighed, after which the mean weight diameter of the soil aggregates (MWD), the geometric mean diameter of the soil aggregates (GMD), and the percentage of >0.25 mm aggregate (R_0.25_) were determined. The proportion of soil aggregates was calculated using the following method [64]:

(13) $$MWD=\frac{\sum_{i=1}^{n}M_{i}\times X_{i}}{\sum_{i=1}^{n}M_{i}}$$

(14) $$GMD=exp\left[\frac{\sum_{i=1}^{n}M_{i}\times lnX_{i}}{\sum_{i=1}^{n}M_{i}}\right]$$

(15) $$R_{0.25}=\frac{M_{>0.25}}{\sum_{i=1}^{n}M_{i}}\times 100\%$$

where MWD is the mean weight diameter, GMD is the geometric mean diameter, R_0.25_ is the proportion of >0.25 mm soil aggregates, M > 0.25 indicates the mass of >0.25 mm soil aggregates, Mi is the mean diameter of degree i aggregates, and X_i_ is the mass of stage i aggregates.

### 4.4. Statistical Analysis

The tables show averages in triplicate. A two-way analysis of variance of treatment, the year, and their interactions were calculated. For multiple comparison tests, the comparison of means was analyzed using Tukey’s test at *p* < 0.05 with SPSS 26.0 (IBM SPSS Inc., Somers, NY, USA). Origin 2021 (Origin Lab, Corporation, Northampton, MA, USA) was used for plotting, and the crop yield, root distribution, and soil physical and chemical properties were normalized by principal component analysis (PCA).

## 5. Conclusions

Rice under dry cultivation–maize intercropping improved the system productivity and land-use efficiency by regulating the coordination of interspecific promotion and competition. The three-year average LER of the intercropping was 1.05, and the intercropping increased the maize yield compared to monocropping, which was attributed to the fact that the intercropped maize exhibited a higher A_MR_ (2.50) and K_MR_ (1.56) as the dominant species in the co-growth period. The intercropped maize root system showed an obvious asymmetric distribution, which favored the root uptake of soil nutrients and water. This intercropping system reduced the soil bulk density and increased the soil porosity, MWD, GMD, the proportion of >0.25 mm aggregates in the 0–30 cm soil layer, and SOM content in the 10–20 cm soil layer. The rice under a dry cultivation–maize intercropping system improved the soil environment by promoting belowground root growth, which in turn, improved the yield and land-use efficiency.

## Figures and Tables

**Table 1:** Yields of rice under dry cultivation and maize, the total yield, and the land-equivalent ratio (LER) for rice under dry cultivation–maize intercropping in three years.

Year	Cropping System	Rice Under Dry Cultivation (kg ha^−1^)	Maize (kg ha^−1^)	Total Yield (kg ha^−1^)	PLER_R_	PLER_M_	LER
2021	Intercropping	5808.95 b	14,580.16 a	10,194.53 a	0.43	0.63	1.06
	Monocropping	6756.48 a	11,486.34 b	9121.02 b			
2022	Intercropping	6527.58 b	12,370.84 a	9449.26 a	0.46	0.61	1.07
	Monocropping	7032.09 a	10,155.82 b	8593.99 b			
2023	Intercropping	6916.80 b	11,831.34 a	9374.04 a	0.47	0.56	1.03
	Monocropping	7422.69 a	10,570.01 b	8995.47 b			
Year		NS	**				
Cropping system		***	***				
Year × Cropping system		NS	NS				

Note: LER, land-equivalent ratio; PLER_R_ and PLER_M_ are partial LERs for the rice under dry cultivation and maize. Different lowercase letters in the same column indicate significant differences between intercropping and monocropping for the same year. ***, *p* < 0.001; **, *p* < 0.01; NS, not significant variance.

**Table 2:** Yield components of rice under dry cultivation and maize in different cropping systems in three years.

Year	Cropping System	EN (# m^−2^)	KN (# ear^−1^)	TKW (g)
2021	IR	414.31 b	64.33 b	23.68 a
	SR	430.01 a	68.67 a	24.11 a
2022	IR	422.11 b	66.83 b	24.36 a
	SR	433.73 a	70.93 a	23.60 a
2023	IR	426.46 b	74.97 a	23.26 a
	SR	435.67 a	76.12 a	23.69 a
2021	IM	5.27 a	625.11 a	336.05 a
	SM	5.29 a	618.40 b	322.27 b
2022	IM	4.77 a	743.41 a	286.53 a
	SM	4.74 a	651.29 b	276.81 b
2023	IM	4.78 a	724.88 a	281.45 a
	SM	4.73 a	704.57 b	269.56 b

Note: IR, intercropped rice under dry cultivation; SR, sole rice under dry cultivation; IM, intercropped maize; SM, sole maize. EN, the ear (or cob) number per square meter; KN, the kernel number per ear (or cob); TKW, the thousand-kernel weight. #, the number. Values followed by lowercase letters within a column indicate significant differences between cropping methods for the same crop for a particular year at the 5% level.

**Table 3:** Aggressivity (A), relative crowding coefficient (K), time–area-equivalent ratio (ATER), and competition ratio (CR) for rice under dry cultivation–maize intercropping over the three years.

Year	A			K			ATER	CR_MR_
	A_M_	A_R_	A_MR_	K_M_	K_R_	K_MR_		
2021	2.54	1.57	0.96	1.61	0.62	1.00	1.27	1.61
2022	2.44	1.72	0.72	1.48	0.74	1.10	1.16	1.42
2023	2.52	1.73	0.79	1.58	0.75	1.18	1.16	1.46

Note: A, the index of aggressivity, K, the crowding coefficient relative, A_M_, the aggressive of corn, A_R_, the aggressive of rice under dry cultivation, A_MR_, aggressiveness coefficient of maize relative to rice under dry cultivation, K_M_, crowding coefficient of corn, K_R_, crowding coefficient of rice under dry cultivation, K_MR_, crowding coefficient of maize relative to rice under dry cultivation, ATER, the area–time equivalence ratio; CR_MR_, the competitive ratio of the maize relative to the rice under dry cultivation.

**Table 4:** The accumulation of N, P, and K in aboveground plants in rice under dry cultivation and maize in different cropping systems over two years (kg ha^−1^).

Year	Treatment	N	P	K
2022	IR	92.12 b	16.12 b	89.28 b
	SR	106.54 a	24.05 a	117.21 a
	IM	194.65 a	42.55 a	205.14 a
	SM	165.73 b	34.29 b	172.15 b
2023	IR	106.51 b	26.53 b	103.20 b
	SR	125.70 a	34.50 a	138.22 a
	IM	180.58 a	66.88 a	312.40 a
	SM	147.79 b	52.51 b	272.49 b

Note: IR, intercropped rice under dry cultivation; SR, sole rice under dry cultivation; IM, intercropped maize; SM, sole maize. Values followed by lowercase letters within a column indicate significant differences between cropping methods for the same crop for a particular year at the 5% level.

## Data Availability

Data will be made available on request.
